# Description of normal head structures of the one-humped camel (*Camelus dromedarius*) by magnetic resonance imaging, computed tomography, and cross-sectional anatomy

**DOI:** 10.14202/vetworld.2020.1581-1587

**Published:** 2020-08-14

**Authors:** Hassan Emam, Mohamed Aref, Ahmed Abdelbaset-Ismail, Ahmed Abdelaal, Shaimaa Gouda, Mohamed Gomaa

**Affiliations:** 1 Department of Anatomy and Embryology, Faculty of Veterinary Medicine, Zagazig University, 44159 Zagazig, El-Sharkia, Egypt; 2 Department of Surgery, Radiology and Anaesthesiology, Faculty of Veterinary Medicine, Zagazig University, 44159 Zagazig, El-Sharkia, Egypt; 3 Department of Animal Medicine, Faculty of Veterinary Medicine, Zagazig University, 44159 Zagazig, El-Sharkia, Egypt

**Keywords:** camel, computed tomography, cross-leveling, head region, magnetic resonance imaging

## Abstract

**Aim:**

This study was designed for the 1^st^ time to describe the normal head structures of one-humped camel (*Camelus dromedarius*) using both magnetic resonance imaging (MRI) and computed tomography (CT) as well as cross-sectional anatomy.

**Materials and Methods:**

Five fresh cadaver heads were collected from clinically normal camels and then subjected to T1-weighted MR and CT imaging. Afterward, these examined heads were transversely sliced to obtain seven crossing levels.

**Results:**

The obtained structures per each crossing level were matched with their relevant sorted images of T1-weighted MRI and CT, then identified and labeled accordingly.

**Conclusion:**

The data shown herein expand our knowledge of the normal head structures of the camel and could be used as a reference for ultimate diagnosis of the surgical affections of head using MRI and/or CT.

## Introduction

In veterinary practice, there are two imaging-based diagnostic techniques that are clinically used to efficiently describe the details of the head region: Magnetic resonance imaging (MRI) and computed tomography (CT) [1-3]. To have that purpose, these techniques have mostly become preferred in head area over ultrasonography and classical radiography, respectively, this mainly due to complexity of the head structures. In addition, the osseous cage “skull” hinders ultrasound waves to reach the inner parts of the head [4-8].

CT is considered the best tool for outlining the details of bone structures, while MRI is specifically appropriate for evaluating the soft tissues [6,7].

In large animals, various literatures have discussed the clinical importance of both MRI and CT for the identification of anatomical details as well as diagnosis of conditions affecting head region [3,9-14].

In camel, there are few studies described the normal nasal and oral cavities by CT [15], normal temporomandibular joint (TMJ) by CT and MRI [16], normal brain and cranioencephalic structures by MRI [6,7,17], normal digits by MRI [18], normal carpus and metatarsophalangeal joints by CT [19,20], and normal head by CT [21,22]. To the author’s knowledge, there are no published data so far describing the normal MRI features of the mature camel head in comparison to their close relevant sectional anatomy.

The purpose of this study was, therefore, to identify the detailed anatomical structures of head in normal dromedary camel and to show their interpretation on MR and CT images.

## Materials and Methods

### Ethical approval

All institutional and national guidelines for the care and use of animals were followed according to the Egyptian Medical Research Ethics Committee (ZU-IACUCF203/2019).

### Study period and location

This study was constructed at Faculty of Veterinary Medicine, Zagazig University, Egypt between June 2018 and December 2019.

### Animals

Five cadaver heads of adult mixed sex Baladi one-humped camel (*Camelus dromedarius*) were used for this study. The age of the camels whose heads were harvested ranged from 3 to 6 years old. The camels’ heads were collected from a public slaughterhouse located in Sharkia Governorate, Egypt. Camels were healthy based on their clinical examination made by Veterinarians at the abattoir. The heads were separated at the site of the atlanto-occipital articulation, kept cooled, and subjected to scanning within 4 h post-harvesting.

### CT imaging

CT scanning was made using helical CT scanner of Hispeed NX/I Dual Slice CT (GE, Japan). During scanning, the head was ventrally positioned and the images were captured in transverse plane. The scanning exposure factors used were 120 kV (anode voltage) and 200 mA (electrical current intensity). In addition, the window width and window level were adjusted to obtain better visualization of bone and soft tissues. The processing software V6.1 (GE, Japan) was then used to optically discriminate between bone and soft tissues. In addition, the CT slices were objectively sorted at seven anatomical levels; the first at lateral nasal alae of nostril, the second at first canine tooth, the third at intermandibular symphysis, the fourth at middle nasal region (at 2^nd^ cheek teeth), the fifth at caudal nasal region (at 3^rd^ cheek teeth), the sixth at rostral orbital region (at 4^th^ cheek teeth), and the seventh at middle part of cranial cavity (at TMJ).

### MRI

MR scanning was made using internal Magnetom of 1 tesla field strength (Philips, Intra, USA) and a human body coil. The images were obtained in a transverse plane using fast spin-echo (FSE) T1-weighted sequences. The heads were ventrally positioned during the whole time of scanning. The images were made using 350 ms repetition time (TR), 0.8 s echo time (ET), and one excitation (E). The MRIs were afterward processed and objectively sorted to be at seven anatomical levels similar to that of CT scanning.

### Transverse anatomical sectioning

After completion of CT and MRI, the heads were immediately placed in a formaldehyde solution (4%) for 48 h for fixing the tissues and then stocked at −15°C and were cross-sectionally sliced using an electrical saw at the same matched seven levels. Each anatomic slice was thoroughly cleaned and subjected to photography. Each anatomic slice was then compared to its relevant CT and MR views. The osseous and soft structures on all anatomic slices were identified, interpreted, labeled based on the Nomenclature of Nomina Anatomica Veterinaria [23], and subsequently appointed on the corresponding MR and CT images.

## Results

**Figure-1:**
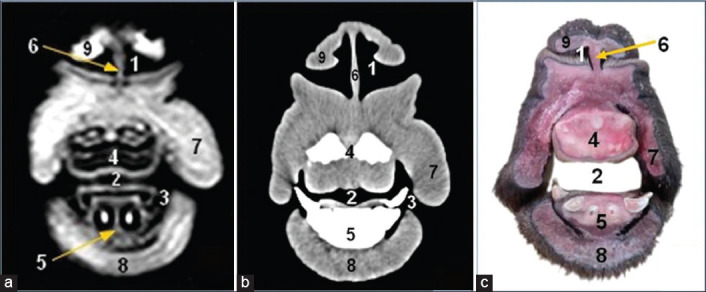
Magnetic resonance image (a), computed tomography image (b), and cross-sectional anatomical image (c) of the camel head at the level of lateral nasal alae of the nostril. 1. Anterior naris; 2. Oral cavity proper; 3. Buccal vestibule; 4. Hard palate; 5. Lower incisors; 6. Cranial end of nasal septum (nerric cartilage); 7. Cheek; 8. Muscle mentalis; 9. Lateral nasal alae containing lateral alar nasal cartilage.

**Figure-2:**
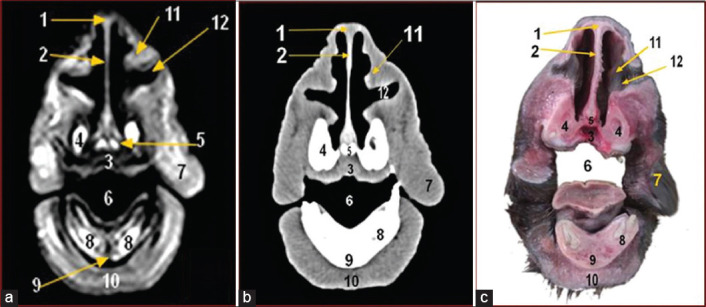
Magnetic resonance image (a), computed tomography image (b), and cross-sectional anatomical image (c) of the camel head at the level of first lower canine tooth. 1. Nasal bone; 2. Nasal septum; 3. Hard palate (palatine process of premaxilla bone); 4. Maxillary bone; 5. Vomer bone; 6. Oral cavity proper; 7. Cheek (muscle buccinator); 8. First lower incisor; 9. Mandible; 10. Muscle mentalis; 11. Dorsal part of ventral nasal concha; 12. Lateral nasal diverticulum.

**Figure-3:**
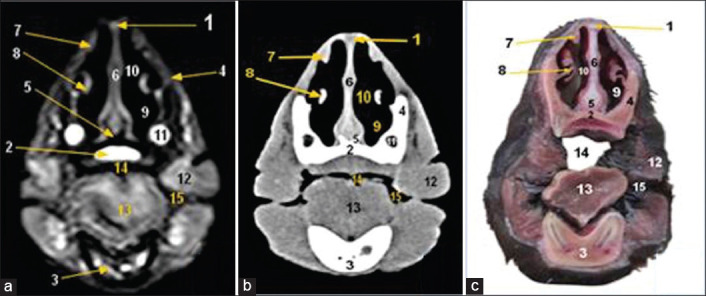
Magnetic resonance image (a), computed tomography image (b), and cross-sectional anatomical image (c) of the camel head at the level of the intermandibular symphysis. 1. Nasal bone; 2. Palatine process of incisive bone; 3. Incisive part of mandible (intermandibular symphysis); 4. Maxillary bone; 5. Vomer bone; 6. Nasal septum; 7. Straight fold of dorsal nasal concha; 8. Dorsal part of ventral nasal concha; 9. Ventral nasal meatus; 10. Middle nasal meatus; 11. Infraorbital canal; 12. Muscle buccinators; 13. Tongue; 14. Oral cavity proper; 15. Buccal vestibule.

**Figure-4:**
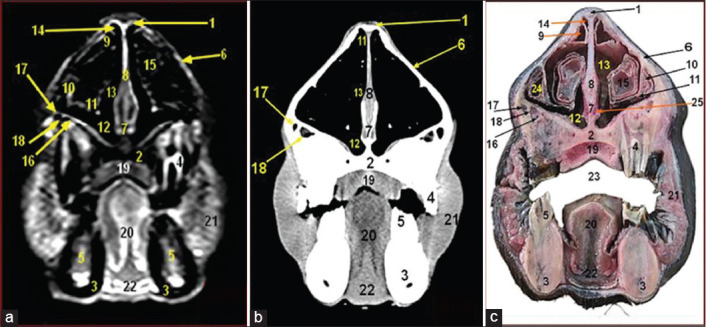
Magnetic resonance image (a), computed tomography image (b), and cross-sectional anatomical image (c) of the camel head at the level of middle nasal region of camel head at the level of the 2^nd^ cheek teeth. 1. Nasal bone; 2. Palatine process of maxilla bone; 3. Molar part of mandible; 4. Upper 2^nd^ cheek tooth; 5. Lower 2^nd^ cheek tooth; 6. Maxillary bone; 7. Vomer bone; 8. Nasal septum; 9. Dorsal conchal sinus; 10. Dorsal part of ventral nasal concha; 11. Ventral part of ventral nasal concha; 12. Ventral nasal meatus; 13. Middle nasal meatus; 14. Dorsal nasal meatus; 15. Middle conchal sinus; 16. Maxillary sinus; 17. Lacrimal sinus; 18. Maxillolacrimal opening; 19. Hard palate; 20. Tongue; 21. Cheek (muscle buccinator); 22. Genioglossus muscles; 23. Oral cavity proper; 24. Ventral conchal sinus; 25. Vomeronasal organ.

**Figure-5:**
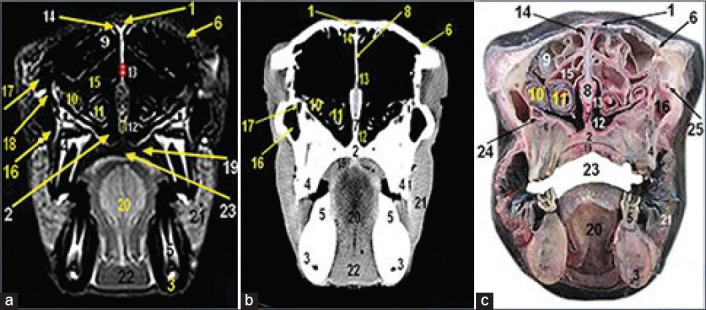
Magnetic resonance image (a), computed tomography image (b), and cross-sectional anatomical image (c) of the camel head at the level of caudal nasal region of camel head at the level of the 3^rd^ cheek teeth. 1. Nasal bone; 2. Palatine process of maxilla bone; 3. Molar part of mandible; 4. 3rd upper cheek tooth; 5. 3rd lower cheek tooth; 6. Maxillary bone; 7. Vomer bone; 8. Nasal septum; 9. Dorsal conchal sinus; 10. Dorsal part of ventral nasal concha; 11. Ventral part of ventral nasal concha; 12. Ventral nasal meatus; 13. Middle nasal meatus; 14. Dorsal nasal meatus; 15. Middle conchal sinus; 16. Maxillary sinus; 17. Lacrimal sinus; 18. Maxillolacrimal opening; 19. Hard palate; 20. Tongue; 21. Cheek (muscle buccinator); 22. Genioglossus muscles; 23. Oral cavity proper; 24. Nasomaxillary opening; 25. Nasolacrimal canal.

**Figure-6:**
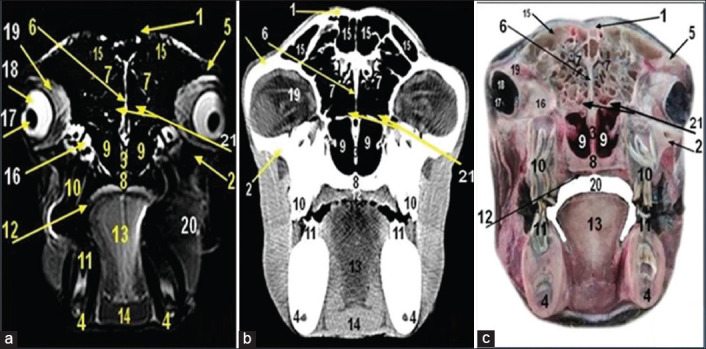
Magnetic resonance image (a), computed tomography image (b), and cross-sectional anatomical image (c) of the camel head at the level of rostral orbital region of camel head at the level of the 4^th^ cheek teeth. 1. Frontal bone; 2. Zygomatic bone; 3. Vomer bone; 4. Molar part of mandible; 5. Zygomatic process of frontal bone; 6. Perpendicular plate of ethmoid bone; 7. Ethmoidal labyrinth; 8. Palatine process of maxilla bone; 9. Choanae; 10. 4^th^ upper cheek tooth; 11. 4^th^ lower cheek tooth; 12. Hard palate; 13. Tongue; 14. Genioglossus and geniohyoideus muscles; 15. Frontal sinus; 16. Periorbital fat; 17. Eye lens; 18. Vitreous chamber of the eye; 19. Extraocular muscles; 20. Oral cavity proper; 21. Sphenoidal sinus.

**Figure-7:**
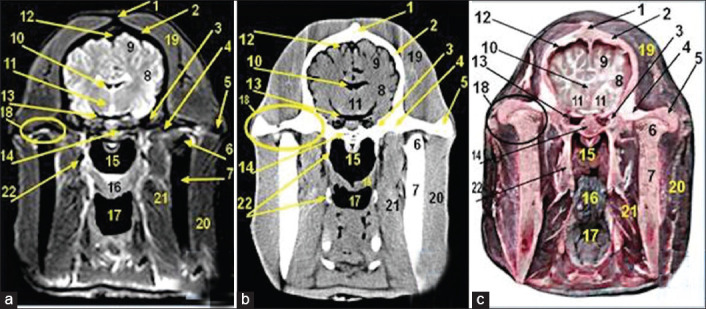
Magnetic resonance image (a), computed tomography image (b), and cross-sectional anatomical image (c) of the middle part of cranial cavity at the level of the temporomandibular joint (TMJ). 1. External sagittal crest; 2. Parietal bone; 3. Squamous part of temporal bone; 4. Retro articular process of temporal bone; 5. Zygomatic process of temporal bone; 6. Condyloid process of mandible; 7. Coronoid process of mandible; 8. Temporal lobe of brain; 9. Parietal lobe of brain; 10. Lateral ventricle; 11. Mesencephalon tectum: Rostral colliculus; 12. Dorsal sagittal sinus of dura mater; 13. Ventral cavernous sinus of dura mater; 14. Body of basisphenoid bone; 15. Nasopharynx; 16. Soft palate; 17. Oropharynx; 18. TMJ; 19. Temporalis muscle; 20. Masseter muscle; 21. Pterygoideus muscle; 22. Stylohyoid bone.

In this study, the images of anatomy, CT, and MRI were arranged per each anatomical level in seven figures (Figures-1-7). The head of camel was arranged into three regions: Nasal, orbital, and cranial regions to make anatomical morphogenesis map of the nasal, orbital, and cranial cavities with their contents, in addition to structures of eye, paranasal sinuses, and brain. The investigation of the head of camel was carried by gross cross-sectional anatomical studies, MRI, and CT scan.

MRI provided good discrimination between bone and soft tissue and moderate discrimination between the adjacent soft tissues according to their physical density difference. Herein, we found that on MRI, the mineral rich tissues (bones and teeth) appeared dark (no signal). Cartilages and muscles appeared gray (low signal intensity, hypointense), while fat and bone marrow appeared bright (high signal, hyperintense). The air containing sinuses and conchae appeared dark without signals(Figures-1-7; Panel A).

Concerning the CT images interpretation, the higher density structures (e.g., osseous tissues) appeared white; air (e.g., paranasal sinuses and conchae) and gases appeared black with no density, whereas intermediate density of soft tissues (e.g., head muscles) appeared gray(Figures-1-7; Panel B).

The anterior nares were surrounded by two nasal alae (lateral and medial). The lateral one contained lateral alar nasal cartilage which presents only in camel (Figure-1).

This study illustrated various anatomical structures of the nasal cavity. The dorsal nasal concha (Figure-4) had a straight fold (Figure-3), the middle one (Figure-4) appeared communicated with nasal cavity while the ventral one (Figures-3-5) was formed from dorsal part (Figure-2) and other small ventral part (Figure-4).

The dorsal and middle meatuses were narrower than ventral one that leads to pathway of the posterior nares (choanae) in addition to, the middle meatus was divided into upper and lower pathways due to the absence of middle nasal concha (Figures-3-5).

The cranial end of nasal septum (Figures-2-5) extended rostrally by the nerric cartilage (Figure-1).

The conchal sinuses appeared caudally within dorsal nasal concha (Figures-4 and 5) which communicated with middle nasal meatus, middle nasal conchal sinuses (Figures-4 and 5) lateral to caudal part of ventral nasal concha while the ventral conchal sinuses (Figure-4) appeared at level of upper cheek tooth with the presence of some recess and bullae.

The vomeronasal organ (Figure-4) appeared as two elongated tubes at ventral nasal meatus supported by a plate of cartilage while the lateral nasal diverticulum appeared as a small pouch ventral to dorsal part of ventral nasal concha (Figure-2). The infraorbital canal (Figure-3) was a canal that extended within maxilla bone. The nasolacrimal canal (Figure-5) extended on the lateral surface of maxilla opened at mucocutaneous junction.

There were five paranasal sinuses in camel head: Frontal, maxillary, sphenoidal, lacrimal, and ethmoidal sinuses. The segmented frontal sinus (Figure-6) extended caudally dorsal to cranial cavity and rostrally to the level of 4^th^–5^th^ upper cheek tooth.

The maxillary sinus (Figures-4 and 5) extended to the level of the rostral border of the 2^nd^, 3^rd^, and 4^th^ upper cheek tooth. It communicated with the lacrimal sinus by maxillolacrimal opening at level of the 2^nd^–3^rd^ upper molar cheek tooth and communicated with middle nasal meatus by nasomaxillary opening. The lacrimal sinus was found rostromedial to the orbital cavity (Figure-4).

The sphenoidal sinus appeared at the level of the 5^th^ upper molar cheek tooth while the ethmoidal sinus appeared within the ethmoidal labyrinth at the level of the 5^th^ upper molar cheek tooth (Figure-6).

The TMJ (Figure-7) formed in between temporal and mandibular condyles with the presence of articular disk.

In the rostral part of the cranial cavity, two olfactory bulbs were present within the ethmoidal fossa of ethmoid bone. The brain has temporal and parietal lobes with dorsal longitudinal fissure in between two cerebral hemispheres. Several brain structures were also identified (Figure-7).

## Discussion

We should first refer to that there are no published data showing the MRI examination of normal head structures of the camel. Thus, we show for the 1^st^ time the normal structures and their appearance of the camel’s head by MRI and these data were interpreted in combination with relevant findings of CT and cross-sectional anatomy.

It is important to clear that the head of camel, similar to that of other animals, is a complex anatomical compartment that hinders the purpose of the physical and clinical examination [12,24]. Despite the imaging of bone and soft tissues of head could be obtained by classical radiography and ultrasonography, respectively, the presence of many bone superimpositions and osseous cage “skull” makes their proper assessment difficult due to lack of good resolution and proper contrast [25].

For these reasons, imaging by CT, as a sectional diagnostic tool, provides superior resolution and excellent differentiation between the osseous and soft tissues, and is, therefore, beneficial to properly discriminate the normal and diseased structures within the head region [26].

As well, MRI, as an excellent diagnostic imaging technique that provides good definition and tissues discrimination, is an excellent tool for accurate assessment of the soft tissues located within the head region using several anatomical planes [27].

In this study, the cadaver heads of the camel were used for CT and MRI examination due to lack of their large fitting Magnetom that contain the size of the camel [6,24]. In addition, the excellent resolution could not be obtained due to the reason of lack of antennas specific for camels [19]. This explains why the MR and CT images were not of sharp spatial definition. Nonetheless, the FSE T1-weighted and CT parameters employed herein to obtain these cross-spatial images can be referenced for subsequent studies on head of the camel.

Since the transverse planes are being beneficial to obtain the anatomic relationships easily, this is the reason of why we focused in this study to show the transverse sections labeled with their respective gross anatomical sections [16].

It was essential to us to perform cross-sectional anatomy to understand and optimize the normal anatomical head structures of the camel that appeared on both MR and CT images. Thus, using this presented anatomical map, the diagnosis of head disorders or abnormalities such as tumors, fractures, and swellings could be readily reached and to avoid misinterpretation, since the head of the camel is to somehow anatomically different when compared with other large animals [21].

## Conclusion

This study provides for the 1^st^ time the anatomical information of the normal head of the dromedary camel by MRI and expands our knowledge about the head structures by CT as well. For camel head region, CT and MRI (FSE T1 weighted) are valuable imaging options for the assessment of the osseous tissues and for optimal discrimination of the soft tissues, respectively. Since the current research has not been able to investigate the potential impact of camel breed as well as sex and age within each breed on the anatomical changes, further research studies could outline that point would be worthwhile. This study would be useful as an assisting reference, particularly when the MRI and CT devices will be used in camel practice in the future.

## Authors’ Contributions

HE and MA conceptualized the study, conducted the anatomic description, and wrote the manuscript. AA-I and MG designed the work and were also responsible for MRI and CT analysis, wrote and edited the manuscript. AA and SG advised the work design, helped in figures preparation, and edited the draft. All authors have read and approved the final version of the manuscript.

## Competing Interests

The authors declare that they have no competing interests.

## Publisher’s Note

Veterinary World remains neutral with regard to jurisdictional claims in published institutional affiliation.
